# Impact of Dysfunctional Adipose Tissue Depots on the Cardiovascular System

**DOI:** 10.3390/ijms232214296

**Published:** 2022-11-18

**Authors:** Rossella D’Oria, Valentina Annamaria Genchi, Cristina Caccioppoli, Isabella Calderoni, Nicola Marrano, Giuseppina Biondi, Anna Borrelli, Ludovico Di Gioia, Francesco Giorgino, Luigi Laviola

**Affiliations:** Department of Precision and Regenerative Medicine and Ionian Area, Section of Internal Medicine, Endocrinology, Andrology and Metabolic Diseases, University of Bari Aldo Moro, 70124 Bari, Italy

**Keywords:** obesity, cardiovascular risk, epicardial adipose tissue, lifestyle modification, GLP-1R agonists, SGLT2 inhibitors, bariatric surgery

## Abstract

Obesity with its associated complications represents a social, economic and health problem of utmost importance worldwide. Specifically, obese patients carry a significantly higher risk of developing cardiovascular disease compared to nonobese individuals. Multiple molecular mechanisms contribute to the impaired biological activity of the distinct adipose tissue depots in obesity, including secretion of proinflammatory mediators and reactive oxygen species, ultimately leading to an unfavorable impact on the cardiovascular system. This review summarizes data relating to the contribution of the main adipose tissue depots, including both remote (i.e., intra-abdominal, hepatic, skeletal, pancreatic, renal, and mesenteric adipose fat), and cardiac (i.e., the epicardial fat) adipose locations, on the cardiovascular system. Finally, we discuss both pharmacological and non-pharmacological strategies aimed at reducing cardiovascular risk through acting on adipose tissues, with particular attention to the epicardial fat.

## 1. Introduction

Obesity (defined as body mass index (BMI) ≥ 30 kg/m^2^) represents a heterogeneous multifactorial disease, which has now reached epidemic proportions [1]. The onset of obesity results from a complex interaction between eating habits and physical activity, as well as socioeconomic, environmental, and genetic factors. Obesity increases the risk for developing multiple pathological conditions, including diabetes mellitus [2], cardiovascular disease (CVD) [2,3], multiple types of cancers [4], and musculoskeletal and mental disorders [5,6]. Obesity-associated morbidities negatively affect quality of life and work productivity, and result in increased healthcare costs. Specifically, multiple clinical and epidemiological studies demonstrate a significant link between obesity and CVD, including coronary heart disease (CHD), heart failure (HF), hypertension, cerebrovascular disease, atrial fibrillation (AF), ventricular arrhythmias and sudden cardiac death (SCD) [7].

Adipose tissue is now considered as an endocrine organ that interacts with multiple organs and tissues, including brain, liver, skeletal muscle, heart, and blood vessels. In addition, experimental and clinical evidence suggests that both quality and function of adipose tissue are important, at least as much as its quantity, in determining the overall health and cardiovascular risks of overweight/obese individuals [8].

To understand how the different deposits of adipose tissue can contribute to the onset and progression of CVD, it is useful to briefly summarize the classification and composition of adipose tissue. Fat tissues in the human body can be classified by morphology into white, brown, or beige subsets [9], and by location into two main anatomical depots, visceral adipose tissue (VAT) and subcutaneous adipose tissue (SAT) [10,11]. VAT, in turn, can be mainly classified into intrathoracic and abdominal, and intrathoracic adipose tissue can be further classified as epicardial adipose tissue (EAT) and pericardial adipose tissue (PAT) [12]. EAT represents the adipose tissue layer that lies between the surface of the heart and the visceral pericardium; PAT is the adipose tissue layer located on the external surface of the parietal pericardium [13]. Multiple cellular and molecular studies have shown how these adipose tissue depots differ in their proteomic and transcriptomic profiles, and numerous epidemiological studies have demonstrated both the correlation between VAT and cardiometabolic risk, and the neutral role of SAT [11,14,15,16].

About one third of the cells within adipose tissue are represented by adipocytes, which are responsible for energy storage in the form of triglycerides. Moreover, adipose tissue contains many other different types of cells, including fibroblasts, endothelial cells, macrophages, stromal cells, immune cells, and preadipocytes. These heterogeneous cell populations secrete a wide range of bioactive products, including cytokines, chemokines, hormones, microvesicles, inorganic molecules and reactive oxygen species (ROS), that exert autocrine, paracrine and/or endocrine effects on neighboring cells or remote tissues and organs. The composition of the secretome released by the cells of the adipose tissue depends on both the localization of the adipose tissue and specific pathological conditions, including inflammation, insulin resistance, and obesity. For example, adipose stem cells (ASC) isolated from the preperitoneal adipose tissue reveal the least pro-inflammatory properties. Conversely, ASC from VAT exhibit the most pro-inflammatory profile, since they secrete the highest levels of IL-6, MCP-1 and G-CSF. Therefore, ASC from visceral and preperitoneal adipose depots could differentially contribute to the chronic inflammatory scenario of obesity [16].

The cardiovascular system is widely affected by the secretome released by adipose tissue. Massive expansion and remodeling of adipose tissue during obesity differentially affects specific adipose tissue depots and may significantly contribute to vascular dysfunction and CVD [17]. Microvessels originating from the remote adipose tissue depots and released into the bloodstream exert their effects on the heart and on the arterial walls. PAT and EAT exert direct effects on the myocardium through paracrine signaling of released bioactive mediators. In addition, intramyocardial lipid accumulation represents another manifestation of ectopic fat storage that may lead to a local adverse effect [18,19].

The aim of this review is to examine the effects of the different fat depots on the cardiovascular system in the context of obesity, and to discuss how weight loss interventions, including diet, pharmacotherapy, and bariatric surgery, may have an impact on the cardiovascular system.

## 2. Effects of Different Dysfunctional Adipose Tissue Depots on the Cardiovascular System

### 2.1. Remote Adipose Tissue Depots

#### 2.1.1. Intra-Abdominal Adipose Tissue

**Figure 1:**
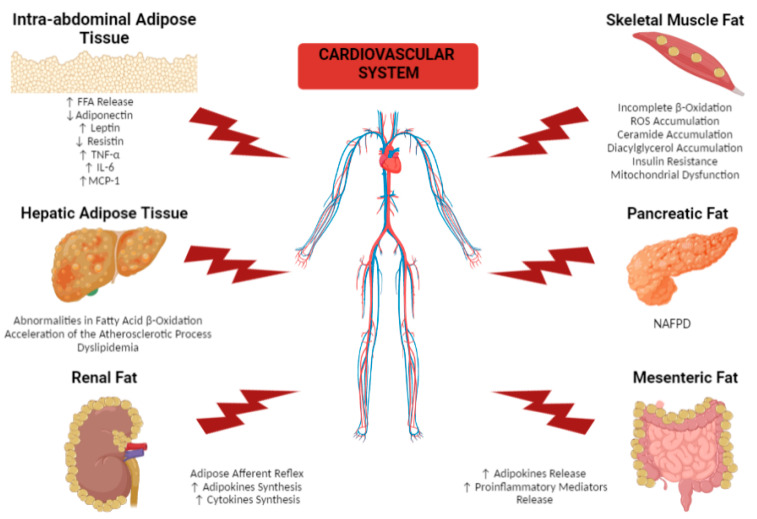
Molecular mechanisms mediating the harmful impact of remote adipose tissue depots on the cardiovascular system in obese patients. The figure illustrates the main molecular mechanisms by which remote fatty acid depots, including intra-abdominal tissue, hepatic adipose tissue, skeletal muscle fat, pancreatic fat, renal fat and mesenteric fat damage the cardiovascular system in obese patients through endocrine and paracrine mediators affecting the heart and the vasculature. FFA, free fatty acid; IL-6, interleukin-6; MCP-1, monocyte chemoattractant protein-1; NAFPD, nonalcoholic fatty pancreas disease; TNF-α, tumor necrosis factor-α; ↑ increased; ↓ decreased.

The intra-abdominal accumulation of visceral fat next to internal organs is associated with an increased risk of clinical CVD (Figure 1) [20]. Visceral fat is characterized by higher lipolytic activity than subcutaneous fat; the consequent excessive release of free fatty acids (FFA) could trigger hepatic insulin resistance, modifications of systemic lipid metabolism, and consequent overt dyslipidemia, leading to increased CVD risk [9].

Other mechanisms by which intra-abdominal adipose tissue might contribute to the onset of CVD include the impairment of molecules secreted by adipocytes, i.e., hormones (adiponectin, leptin, resistin), and proinflammatory cytokines (TNF-α, IL-6, IL-8, and others).

Specifically, adiponectin is an adipokine with beneficial effects on the cardiovascular system, such as reducing inflammatory responses and apoptosis by acting directly on cardiac cells and blood vessels [21]. Adiponectin concentration is down-regulated in patients with obesity-related disorders, including atherosclerosis and ischemic heart disease. Furthermore, the expression of the intra-abdominal adipose tissue-adiponectin receptor 2 (AdipoR2) is reduced in obesity and is inversely correlated with plasma levels of triglycerides. On the other hand, AdipoR1 expression is not impaired in obesity [22].

Leptin is synthesized mainly by adipose cells, but also by other cell types in small quantities [23]. Leptin has been proposed as a key factor in the regulation of body weight and the development of obesity, since it regulates food intake and energy expenditure through a feedback mechanism between the adipose tissue and the satiety center in the hypothalamus [24]. In healthy individuals, leptin also balances blood pressure by modulating the sympathetic activity-dependent vasoconstriction, the endothelial release of nitric oxide, and the angiotensin II-dependent vasoconstriction [25,26]. Conversely, in obese subjects leptin levels are increased, and an organ-specific leptin resistant state has been described [26]. Indeed, despite higher circulating levels, leptin protective vascular effects are attenuated. Notably, leptin physiology is restored after bariatric surgery [27], which is in line with the improvement of the cardiovascular risk factor milieu described post-surgery [28].

Resistin is another adipokine that is secreted mostly by preadipocytes and, to a lesser extent, by mature adipocytes located mostly in the abdomen [29,30]. It promotes endothelial cell activation by inducing endothelin-1 release, upregulates adhesion molecules and chemokines, and downregulates TRAF-3, an inhibitor of CD40 ligand signaling [31]. In addition, resistin induces human aortic smooth muscle cell proliferation, through both the ERK 1/2 and Akt signaling pathways [32]. In obese subjects, plasma resistin levels are increased, and they correlate with markers of inflammation [33]. All of these observations suggest that resistin may be mechanistically linked to CVD in obesity.

TNFα is a pro-inflammatory cytokine that is predominantly produced by monocytes/macrophages. TNFα expression is upregulated in the adipose tissue and in the serum of obese patients, whereas weight loss is associated with a decrease in TNFα levels. TNFα acts on monocytes/macrophages, vascular endothelial cells, and smooth muscle cells to induce the expression of many pro-inflammatory, pro-coagulant, and proliferative genes, contributing to atherosclerosis development [34,35,36,37,38,39]. Specifically, in vascular smooth muscle cells, TNFα induces migration, proliferation, and apoptosis [40,41,42], which are cellular processes of major relevance in vascular diseases. TNFα also induces the rapid expression of adhesion molecules, including E-selectin, vascular cell adhesion molecule-1, and ICAM-1, in endothelial cells [39].

IL-6 is secreted by both adipocytes and macrophages within the adipose tissue [43]. Circulating levels of IL-6 are significantly higher in patients with overweight (BMI range: 25–29.9 kg/m^2^) and obesity (BMI > 30 kg/m^2^), and correlate positively with BMI in patients with grade III obesity, and negatively with serum HDL-C in patients with grade II obesity [44]. Thus, high circulating levels of IL-6 could reflect the intensity of obesity-associated chronic and systemic inflammation, which in turn might contribute to the development of atherosclerosis and CHD, both directly and by reducing HDL cholesterol levels [44]. On the other hand, a different role of IL-6 has been proposed in the context of obesity: in a randomized placebo-controlled trial, abdominally obese adults were treated with tocilizumab, the monoclonal antibody against IL-6 receptor, or with placebo, with either exercise or no exercise [45]. Exercise allows reduction in VAT mass, as well as in body weight and total adipose tissue mass; however, these positive effects of exercise were abrogated in the presence of IL-6 blockade. In addition, in abdominally obese humans, blocking of IL-6 signaling increases VAT extension, total cholesterol, and LDL cholesterol, independently of exercise [45]. These data suggest a dual role of IL-6 in obesity, and potentially relevant metabolic consequences of IL-6 blockade.

Adipocytes and VAT are also able to produce large amounts of monocyte chemoattractant protein 1 (MCP-1), a chemokine directly involved in ventricular remodeling. Indeed, MCP-1 levels are higher in obese patients than in controls and correlate with VAT area, C-reactive protein, and multiple morphological and functional echocardiographic parameters, including left ventricular mass, relative wall thickness, early diastolic filling wave velocity, isovolumetric relaxation time, and deceleration time [46]. In addition, compared with subjects with a lower degree of abdominal adiposity, obese patients with greater amounts of VAT exhibit higher levels of MCP-1 and C-reactive protein [46]. In animal models, MCP-1 deficiency confers protection against the proatherogenic effects of inflammatory VAT [47]. Therefore, MCP-1 may serve as a therapeutic target in obese patients with excessive visceral adiposity and high risk for vascular events.

#### 2.1.2. Hepatic Adipose Tissue

Metabolic dysfunction-associated fatty liver disease (MAFLD), formerly named nonalcoholic fatty liver disease (NAFLD), is a common chronic liver disease with a 70/80% prevalence among obese subjects [48]. MAFLD occurs when ≥5% of the liver cells contain fat and is considered severe when ≥30% of liver cells contain fat on a hepatic biopsy [49]. In addition, increased circulating levels of FFA and plasma amino acids, which occur in obesity, can contribute to the upregulation of inflammatory mediators implicated in the pathogenesis of MAFLD [50]. The pathophysiological and clinical evolution of liver involvement progresses from nonalcoholic steatohepatitis (NASH) to liver fibrosis, cirrhosis, and hepatocellular carcinoma (HCC) [51]. The close association between MAFLD and CVD is supported by observations that CVD is the most common cause of death among MAFLD patients [52]. Abnormalities in liver mitochondrial fatty-acid β-oxidation, underlying MAFLD, are the consequence of a complex network of genes reacting to intracellular and environmental stresses, nutrient overload, and lifestyle habits. The resulting alterations in lipid profile create a pro-atherogenic environment through multiple mechanisms including low-grade inflammation, dyslipidemia, insulin resistance, procoagulant imbalance, hypertension, oxidative stress, fibrosis, and endothelial dysfunction [52,53], which in turn promote cardiovascular complications and increase the risk of cardiovascular mortality in MAFLD patients (Figure 1). Specifically, the strong association between oxidative stress and MAFLD in humans has been suggested by the detection of lipid peroxidation products and 8-hydroxy-deoxyguanosine in the plasma and in liver biopsies from patients with MAFLD [54]. Oxidative stress also plays an important role in the progression from simple steatosis to steatohepatitis [55]. Inflammation is a crucial biological event in the pathogenesis of MAFLD, especially considering recent data showing that adipose tissue is a metabolically active endocrine organ capable of producing proinflammatory cytokines, including TNF-α, IL-6, C-reactive protein, and IL-8 [55]. Insulin resistance promotes lipid accumulation in the liver, which in turn promotes a lack of suppression of endogenous liver glucose production [56]. Therefore, MAFLD further increases insulin resistance and dyslipidemia, causing an acceleration of the atherosclerotic process [57]. Dyslipidemia represents another factor underlying the strong association between CVD and MAFLD: impaired lipid metabolism is associated with upregulation of the transcription factor sterol regulatory element binding protein-1c (SREBP-1c), which is responsible, along with insulin, for the expression of genes involved in de novo lipogenesis, as well as for inhibition of FFA oxidation. The resulting metabolic abnormalities promote an increased hepatic lipid content and an atherogenic lipid profile, ultimately leading to adverse cardiovascular outcomes [58].

#### 2.1.3. Skeletal Muscle Fat

Skeletal muscle adipose depots include perimuscular fat, intermuscular fat, intramuscular fat, paraosseal fat, and intramyocellular lipid accumulation [59]. FFA uptake within the muscle occurs via passive diffusion and through the involvement of three transporters: fatty acid binding protein (FABP), fatty acid transport protein (FATP), and fatty acid translocase (FAT/CD36) [52]. In obese patients, lipid excess overcomes these mechanisms, leading to increased intramyocellular lipid accumulation, incomplete β-oxidation, and ROS accumulation [60] (Figure 1). Intramyocellular lipid accumulation is not lipotoxic per se in obesity, but it could act as a marker for the accumulation of specific lipid species that mediate insulin resistance, including ceramides and diacylglycerols [50] (Figure 1). Specifically, human studies support the relationship between muscle ceramide content and insulin resistance, and muscle ceramide content is shown to be elevated in obese patients compared to lean individuals [61] (Figure 1). In muscle, ceramides trigger insulin resistance through inhibition of the serine/threonine-specific protein kinase Akt/protein kinase B (PKB) [62], which in turn is responsible for GLUT4 translocation to the sarcolemma [63]. Ceramides are also linked to mitochondrial dysfunction, since they interfere with mitochondrial bioenergetics by inhibiting electron transport at complex I and complex III [64], and by elevating ROS production [65] (Figure 1). Conversely, inhibition of ceramide synthesis prevents insulin resistance induced by lipid oversupply [66]. Other studies suggest that lipid excess does not result in increased ceramide content, but increases muscular content of emulsions consisting primarily of unsaturated fatty acids [67,68]. Thus, lipid overload with saturated fatty acids preferentially induces ceramide-mediated insulin resistance, whereas overload with unsaturated lipids may result in diacylglycerol-mediated insulin resistance [62] Interestingly, both muscle diacylglycerol and ceramide content are elevated in obese patients [69]. Elevated diacylglycerol content increases the activity of protein kinase C (PKC) ε and θ [70], which in turn induce serine phosphorylation of insulin receptor substrate 1 (IRS-1), thus inhibiting kinase activity and subsequent activation of PI3-kinase and Akt/PKB [70]. 

Recent clinical studies have directly analyzed the clinical role of skeletal muscle fat in cardiometabolic risk. Specifically, the association of abdominal intermuscular adipose tissue (IMAT) volume with coronary artery calcification was evaluated in participants in the Coronary Artery Risk Development in Young Adults (CARDIA) study [71]. This multicenter study showed that higher abdominal skeletal muscle adipose tissue volume is related with subclinical atherosclerosis independently of traditional CVD risk factors and other adipose depots.

Similarly, results from the SECRET study suggest that regional adipose deposition, along with total body fat, might have important adverse consequences in older obese patients affected by HF with preserved ejection fraction (HFpEF), including exercise intolerance. In addition, specific interventions targeting intra-abdominal and thigh intermuscular fat have proven capable of improving the clinically important outcome of exercise intolerance [72].

#### 2.1.4. Pancreatic Fat

Pancreatic ectopic fat is referred to as interlobular or intralobular infiltration of adipocytes [73] and accumulation of intracellular lipid droplets in pancreatic endocrine or exocrine cells [74,75,76]. Pancreatic fat increases with age, obesity, diabetes mellitus, excess alcohol intake, and viral infections [75,77,78]. Recently, nonalcoholic fatty pancreas disease (NAFPD) was proposed as a pathological condition related to obesity independently from alcohol consumption [79]. Each of the intra-pancreatic fat compartments might be involved in the pathogenesis of type 2 diabetes mellitus (T2DM), CVD, and diseases of the exocrine pancreas [80] (Figure 1). Ozturk et al. confirmed the strong association between NAFLD and fatty pancreas and showed for the first time that fatty pancreas might contribute to the development of atherosclerosis in patients with NAFLD. Moreover, fatty pancreas was shown to be associated with higher BMI, thus also contributing to the development of diabetes and metabolic syndrome [81]. Kul et al. investigated whether NAFPD is associated with EAT and aortic intima-media thickness (aIMT), which is a surrogate marker of subclinical atherosclerosis. Both EAT and aIMT were significantly higher in NAFPD positive subjects, compared to NAFPD negative subjects, suggesting that NAFPD might reflect subclinical atherosclerosis [82]. Recently, Lee et al. retrospectively evaluated risk factors for hypertension in 65 children with NAFLD who underwent liver biopsy and magnetic resonance imaging-based fat fraction measurement for ectopic hepatic and pancreatic fats, as well as anthropometric evaluation, blood pressure assessment, laboratory tests, and body composition analysis. The authors observed that in addition to BMI-z, ectopic pancreatic fat represents an important risk factor for hypertension in children with NAFLD [83]. Further studies are needed to fully elucidate the impact of pancreatic fat on the onset of CVD, as well as the underlying molecular mechanisms.

#### 2.1.5. Renal Fat

Renal fat includes perirenal, pararenal, and renal sinus fat, respectively. Anatomically, perirenal fat is located in the retroperitoneal space surrounding the kidney and is supported by the renal fascia, also called Gerota’s fascia. Pararenal fat is adjacent to perirenal fat and is separated from perirenal fat by the renal fascia. Renal sinus fat is a deposit of perivascular adipose tissue that is located at the medial border of the kidney [84]. Excess para- and perirenal fat has recently been identified as an emerging risk factor for CVD, independent of typical metabolic parameters [85]. Indeed, a recent study evaluated the relationship between perirenal fat and hypertension in a population of 284 morbidly obese patients (BMI ≥ 40 or ≥35 kg/m^2^) and the potential variations after sleeve-gastrectomy. At baseline, the perirenal fat thickness was higher in hypertensive than in non-hypertensive obese subjects (13.6 ± 4.8 vs. 11.6 ± 4.1 mm, *p* = 0.001), and showed a significant direct correlation with age, waist circumference, BMI, systolic blood pressure, insulinemia, HOMA-IR, glycated hemoglobin, and creatinine. After surgery, perirenal fat thickness significantly decreased (from 13 ± 4 to 9 ± 4 mm, *p* < 0.001). Therefore, the perirenal fat thickness in obese patients could be a valuable tool to define the risk of developing hypertension, providing the clinician with an additional parameter to define those who could benefit most from bariatric surgery [85]. Similarly, De Pergola et al. examined whether para- and perirenal fat accumulation is associated with higher 24 h mean systolic (SBP) and/or diastolic blood pressure (DBP) levels in overweight and obese subjects (BMI: 33.3  ±  4.3 kg/m^2^). Para- and perirenal ultrasonographic fat thickness (PUFT) was significantly and positively correlated with waist circumference, insulin, HOMA-IR, and 24 h mean DBP levels. A multivariate analysis by multiple linear regression showed that the association of 24 h mean DBP as dependent variable with PUFT (multiple R = 0.34; *p* = 0.026) was independent of other anthropometric, hormonal and metabolic parameters [86]. The relationship of para- and perirenal fat with metabolic parameters in overweight and obese subjects was also analyzed by Manno et al. [87]. In addition, the authors analyzed the relationship between epicardial fat and metabolic parameters. Specifically, epicardial fat thickness (EUFT) was positively associated with age, BMI, waist circumference, systolic and diastolic blood pressure, and LDL-cholesterol. A multivariate analysis by multiple linear regression showed a direct association of waist circumference with both PUFT and EUFT, a correlation of PUFT with HOMA-IR (positive) and HDL-cholesterol (negative), and a direct association of EUFT (both long axis and short axis) with LDL-cholesterol. All of these correlations were independent of other anthropometric, metabolic, and hemodynamic parameters. Given the strong relationship between para- and perirenal fat excess and CVD, the assessment of perirenal fat thickness could be considered an important tool in assessing the cardiovascular risk profile.

#### 2.1.6. Mesenteric Fat

Mesenteric fat is the portion of visceral fat draining into the portal circulation. Among the different types of visceral adipose tissues, mesenteric fat is considered particularly detrimental [88], and, metabolically more active [89]. Multiple factors affect the accumulation of mesenteric fat, including age, sex, sex hormones, genetic factors, ethnicity, nutritional factors, and lifestyle [90]. Recently, mesenteric fat thickness has been associated with metabolic and cardiovascular markers, including blood lipoprotein concentrations and apolipoprotein A-II [91]. Liu et al. examined the independent relationship between mesenteric fat thickness and metabolic syndrome. Mesenteric fat thickness had significant correlations with multiple metabolic variables, and was found to be an independent determinant of all components of metabolic syndrome on multivariate regression. Interestingly, the odds ratio of metabolic syndrome was increased by 1.35 (95% CI 1.10–1.66)-fold for every 1 mm increase in mesenteric fat thickness. A mesenteric fat thickness ≥ 10 mm was the optimal cutoff value to identify metabolic syndrome, since subjects with mesenteric fat thickness ≥ 10 mm had higher carotid IMT than those with thickness < 10 mm (0.73 ± 0.19 vs. 0.64 ± 0.16 mm, *p* = 0.001) [92]. Similar results were obtained in a prospective cohort study, in which the close interrelationships between mesenteric fat thickness, carotid IMT, and CAD in metabolic syndrome patients were demonstrated [93]. Severity and prevalence of CAD were significantly higher in patients with a mesenteric fat thickness of 10 mm or more. Moreover, there was a significant positive correlation between carotid IMT and multiple diseased vessels in patients with CAD. These data suggest that mesenteric fat thickness and IMT are good indicators of the prevalence and severity of CAD in patients with metabolic syndrome.

### 2.2. Cardiac Fat: Focus on Epicardial Adipose Tissue

**Figure 2:**
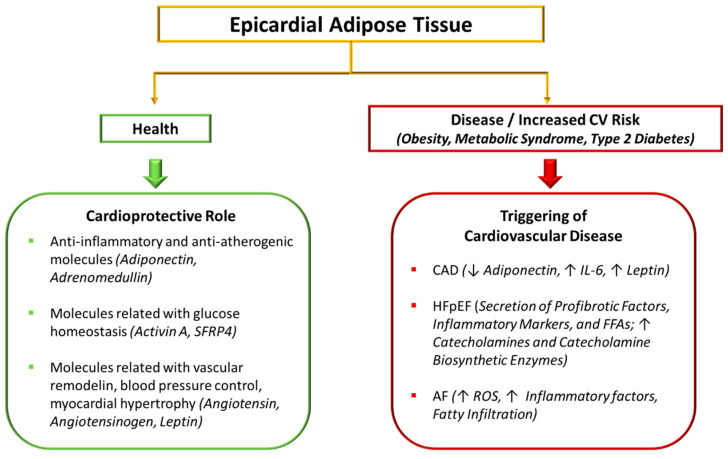
Mechanisms and mediators of beneficial and deleterious effects of epicardial adipose tissue on cardiovascular risk. In healthy conditions, EAT shows protective functions by secreting cardioprotective adipokines, including anti-inflammatory and anti-atherogenic molecules (e.g., adiponectin and adrenomedullin), molecules related with glucose homeostasis (e.g., activin A and SFRP4), and molecules involved in vascular remodeling, blood pressure control, myocardial hypertrophy and adipogenesis (e.g., angiotensin, angiotensinogen, leptin). Under specific pathological conditions, including obesity, metabolic syndrome and type 2 diabetes, the beneficial properties of EAT might be impaired, and EAT might thus promote the development of cardiovascular disease, including CAD, HF, and AF, through multiple molecular mechanisms: reduced expression of adiponectin, increased expression of IL-6 and leptin, secretion of profibrotic factors, inflammatory markers, and FFAs, increase in catecholamine levels and in catecholamine biosynthetic enzymes, augmented ROS production, and fatty infiltration of the atrium. AF, atrial fibrillation; CAD, coronary artery disease; EAT, epicardial adipose tissue; FFAs, free fatty acids; HF, heart failure; ROS, reactive oxygen species; SFRP4, secreted frizzled-related protein 4; ↑ increased; ↓ decreased.

EAT is defined as a fat depot localized between the pericardium and the myocardium [94] that can modulate the biological functions of surrounding tissues through multiple cytokines and chemokines mediating paracrine and vasocrine effects [11,95]. EAT is emerging as a key risk factor for atherosclerosis and cardiovascular events [94,96], and a novel potential therapeutic target in CVDs. In healthy conditions, EAT shows protective functions, including mechanical protection against arterial wave torsion [97] and thermogenesis as a defense against hypothermia [97,98]. In addition, EAT represents an energy source [97,99], and through its ability to rapidly use FFA, EAT may protect the myocardium from the lipotoxic damage of high levels of FFA [100]. Importantly, EAT secretes cardioprotective adipokines, including anti-inflammatory and anti-atherogenic molecules, molecules related with glucose homeostasis, and molecules involved in vascular remodeling, blood pressure control, myocardial hypertrophy and adipogenesis [97] (Figure 2).

Specifically, EAT secretes adiponectin [97], which protects coronary circulation and improves endothelial dysfunction and oxidative stress [101,102], and adrenomedullin [97], which causes vasorelaxation and modulates vascular proliferation by closely interacting with nitric oxide [103]. Conversely, reduced levels of both adiponectin and adrenomedullin are linked with the pathogenesis of coronary artery disease, hypertension, and cardiac and renal failure [103,104].

Activin A belongs to the transforming growth factor beta (TGF-β) family and has an important role both in glucose homeostasis and inflammatory responses [97]. Chen et al. showed that circulating activin A levels are associated with impaired myocardial glucose metabolism and high left ventricular mass/volume-ratio in patients with uncomplicated type 2 diabetes, thus reflecting a potential detrimental role in early human diabetic cardiomyopathy [105]. In addition, an anti-inflammatory potential of activin A has been suggested in patients with angina [106]. Secreted frizzled-related protein 4 (SFRP4) is an extracellular regulator of the wingless-type mouse mammary tumor virus integration site family (WNT) pathway, and it has been implicated in adipocyte dysfunction, obesity, insulin resistance, and impaired insulin secretion in patients with type 2 diabetes [107]. Interestingly, EAT-derived and circulating SFRP4 expression levels are increased in patients with coronary artery disease (CAD) [108].

EAT-released adipokines associated with vascular remodeling, blood pressure control myocardial hypertrophy and adipogenesis are angiotensin, angiotensinogen, and leptin [97]. The expression levels of these adipokines are increased in CAD patients [109], thus suggesting that, under specific pathological conditions, including obesity, metabolic syndrome, chronic inflammation and type 2 diabetes, the beneficial properties of EAT might be impaired [110,111]. All of these factors could contribute to the development of CVD because of the anatomical proximity of EAT with coronary arteries and myocardium [112] (Figure 2).

Specifically, deleterious effects of EAT on metabolic and cardiovascular functions include secretion of proinflammatory and proatherogenic EAT-secreted adipokines and cytokines (i.e., TNF-α, IL-6, and MCP-1), with consequent amplification of vascular inflammation and plaque instability through apoptosis and neovascularization [95], as well as damage mediated by lipotoxicity through myocardial fatty acid infiltration [97].

The relationship between adipose tissue and CAD is highlighted by multiple factors, including the alteration in the adipokine pattern from EAT in CAD patients, the proinflammatory phenotype of EAT in patients affected by CAD, and the correlation among the amount of EAT, its proinflammatory state, and the severity of CAD, as well as the instability of the atherosclerotic plaques [95,113,114,115,116,117]. Iacobellis et al. demonstrated for the first time that human EAT expresses adiponectin, which was found to be significantly lower in EAT isolated from patients with CAD as compared with controls [115]. In addition, Eiras et al. determined that the extension of CAD is significantly associated with the expression of adiponectin and IL-6 mRNA in EAT, suggesting that low adiponectin and high IL-6 expression by EAT may contribute to CAD severity [114]. In patients with CAD, the EAT is also characterized by the secretion of higher leptin levels than in control subjects [116]. Leptin is an adipokine known to play a role in multiple biological processes, including regulation of energy homeostasis, inflammation, vascular function, and angiogenesis [118]. While physiological concentrations of leptin may exhibit multiple beneficial effects, the hyperleptinemia that is typically found in obesity and diabetes is a major risk factor for the development of atherosclerosis, since it exerts potent proatherogenic effects on multiple vascular cell types (macrophages, endothelial cells, and smooth muscle cells) [119]. These effects can also be mediated via an interaction of leptin with the long form of leptin receptor, abundantly expressed in atherosclerotic plaques [117].

Dysfunctional EAT is also involved in the development of HF. Approximately 50% of HF patients have HFpEF, a pathological condition that occurs when left ventricular filling and relaxation are affected, but a good systolic function is preserved. HFpEF represents the most common myocardium disorder among obese patients [120]. EAT has been suggested to play a role in HF, particularly in patients with HFpEF [121,122,123,124], through inflammation, fibrosis, and neural dysregulation [125]. Specifically, the EAT proteome can contribute through paracrine secretion of profibrotic factors, including α1-antichymotrypsin (ACT; also known as serpin A3) and matrix metalloproteinase 14 (MMP14) [126], inflammatory markers such as p53 mRNA [127], and FFA [128]. In addition, large and fibrotic EAT can also exert mechanical effects on both diastolic and systolic function [128]. EAT also showed an enhanced adrenergic activity in patients with HF, demonstrated by increased catecholamine levels and the expression of catecholamine biosynthetic enzymes. These events might contribute to the increased prevalence of AF in patients with HFpEF [129]. In a meta-analysis of 22 studies, Nerlekar et al. showed a significant correlation between increasing EAT mass and the presence of diastolic dysfunction [130]. Interestingly, patients affected by HFpEF and obesity are characterized by a different clinical phenotype than nonobese HFpEF subjects. Obese HFpEF patients display increased plasma volume, more concentric left ventricular remodeling, greater right ventricular dilatation, more right ventricular dysfunction, increased epicardial fat thickness, and greater total epicardial heart volume, despite lower N-terminal pro-B-type natriuretic peptide levels [131]. In addition, compared with nonobese HFpEF and control subjects, obese patients with HFpEF show worse exercise capacity, higher biventricular filling pressures with exercise, and depressed pulmonary artery vasodilator reserve. Koepp et al. analyzed patients with the obese phenotype of HFpEF and classified them based on increased EAT thickness (EAT thickness ≥ 9 mm) [132]. Among patients with the obese phenotype of HFpEF, the presence of increased EAT is associated with more profound hemodynamic derangements, including greater elevation in cardiac filling pressures, more severe pulmonary hypertension, and greater pericardial restraint, culminating in poorer exercise capacity [132]. Further studies are needed to understand the biology of, and identify specific therapies for, excessive EAT in patients with HFpEF, and the potential role of novel therapeutical approaches targeting weight loss in the treatment of HFpEF.

AF represents the most common form of arrhythmia in the adult population. Multiple factors contribute to the development of AF, including hypertension [133], valvular diseases [134], oxidative stress and inflammation [135,136,137]. Multiple mechanisms link EAT with AF, including inflammatory factors, ROS released from EAT, and fatty infiltration of the atrium. Recently, Liu et al. assessed the relationship between EAT levels of interleukin-1β (IL-1β) and persistent AF [138]. Both EAT thickness and mRNA levels of IL-1β were significantly greater in patients with persistent AF than in the control group. In contrast, adiponectin expression was shown to be lower in persistent AF patients [138]. All of these findings suggest that increased levels of regional IL-1β in EAT are associated with persistent AF. ROS play an important role in the pathogenesis of AF, since they are involved in producing arrhythmic substrates [139]. In fact, EAT is characterized by higher oxidative stress activity when compared with SAT in patients with CVD, since proteomic analysis revealed post-translational modifications of antioxidant enzymes and lower catalase expression in EAT as compared with SAT [140]. In addition, Haemers et al. suggested that AF is associated with the fibrosis of subepicardial fatty infiltrates, which contributes to the progressive fibrosis of subepicardial areas of the atrial myocardium [141]. Impaired secretion of cytokines and excessive fatty infiltration may thus represent potential pathological mechanisms linking EAT with AF [126].

## 3. Strategies for Reducing Cardiovascular Risk That Impact Adipose Tissue

### 3.1. Lifestyle Modifications: Restricting Food Intake and Increasing Energy Expenditure

In overweight or obese subjects, weight loss through restriction of food intake or increases in energy expenditure represents the first intervention to reduce the severity of CVD risk. To demonstrate the beneficial effects of diet, an interventional study was performed in 20 severely obese subjects (12 women, 8 men; age 35 ± 10 years; BMI 45 ± 5 kg/m^2^) who underwent a 6-month very low-calorie diet weight loss program. At the end of the study, body weight was found to be reduced by 20%, BMI by 19%, and waist circumference by 23% as compared with baseline values. Importantly, epicardial fat thickness also significantly decreased, by 32%, and modifications in epicardial fat parameters were significantly associated with obesity-related cardiac morphological and functional changes during weight loss [141]. Similarly, in another study, 27 moderately obese men (age 45.8 ± 1.7 years; BMI 30.5 ± 0.7 kg/m^2^) followed a daily low-calorie diet as part of a clinical 12-week weight loss interventional study. The EF thickness was evaluated by transthoracic echocardiography, the abdominal fat tissues by computed tomography scans, and the regional and whole body fat compartments by dual X-ray absorptiometry. An average decrease of 26.8% in calorie intake was associated with post-program reductions of 17.2%, 11.0%, 16.6%, and 29.8% in EF thickness (*p* < 0.001), body mass, percentage fat mass, and abdominal fat compartments, respectively. The percentage change in VAT in response to weight loss was twice as high as the change in EF tissue (*p* < 0.001) [142]. Altogether, these results support the idea that moderate diet-induced weight loss might represent an effective nonpharmacological strategy for reducing EF, which represents a pathogenic fat depot and an emerging marker of VAT.

The effects on adipose tissues of weight loss strategies based on physical exercise have been investigated in multiple trials, with mixed results. Borges et al. tested the effects of weight loss, with and without exercise training (aerobic or resistance), on intra-abdominal adipose tissue and risk factors for CVD in a randomized controlled trial [143]. Specifically, 122 overweight premenopausal women (BMI: 27–30 kg/m^2^) were randomly assigned to one of three groups, i.e., diet only, diet and aerobic training, or diet and resistance training, with a goal of BMI < 25 kg/m^2^. The authors concluded that diet, alone or combined with aerobic or resistance training, resulted in similar improvements in IAAT and CVD risk factors after weight loss, thus supporting the notion that reaching a BMI of less than 25 kg/m^2^ is associated with reduced visceral fat and improved CVD risk [143]. On the other hand, multiple epidemiological studies and systematic reviews have shown that the specific reduction in the thickness of EAT obtained with physical exercise is associated with positive effects on cardiovascular risk factors [144]. Since PAT is also emerging as an important risk factor for CVD, there is a growing interest in discovering strategies to reduce the accumulation of fat also in this depot. A randomized controlled trial demonstrated that in individuals with abdominal obesity, both endurance and resistance training reduced EAT mass, while only resistance training reduced PAT mass. Thus, these data highlight the importance of different exercise modalities as a strategy to reduce both cardiac fat and CV risk factors in individuals with abdominal obesity [145]. 

### 3.2. Pharmacotherapy: Focus on GLP-1 Receptor Agonists and SGLT2 Inhibitors

Glucagon-Like Peptide-1 Receptor Agonists (GLP-1RAs) represent injectable therapies for the treatment of type 2 diabetes and provide significant weight loss, resulting from appetite control, increased satiety, and delayed gastric emptying [146]. Additionally, these molecules have shown relevant cardioprotective effects, including improvement of blood pressure, cardiac function and ischemia, and inflammation [147]. Several cardiovascular outcome trials (CVOTs) involving GLP-1RAs have supported the overall cardiovascular benefits of these drugs [148].

The expression of GLP-1R in EAT has recently been analyzed. RNA-sequencing analysis and immunofluorescence carried out by Iacobellis and colleagues clearly showed for the first time that EAT expresses both GLP-1R and GLP-2R genes, thus suggesting that pharmacologically targeting EAT may induce beneficial cardiovascular and metabolic effects [149]. Dozio et al. collected EAT biopsies from CAD patients undergoing coronary artery bypass graft for microarray analysis of GLP-1R and genes involved in FA metabolism and adipogenesis [150]. EAT GLP-1R was directly correlated with genes promoting beta-oxidation and white-to-brown adipocyte differentiation, and inversely with pro-adipogenic genes. In addition, GLP-1 and GLP-2 levels were higher in CAD than in control subjects, and also higher in patients with greater EAT thickness. Thus, it is tempting to speculate that GLP-1 analogs may activate EAT GLP-1R and, therefore, reduce local adipogenesis, improve fat utilization and induce brown fat differentiation. The increased levels of circulating GLP-1 and GLP-2 may be compensatory mechanisms related to CAD and EAT expansion [150]. The ability of the GLP-1 liraglutide to reduce EAT was tested in a 6 month randomized, open-label, controlled study performed in 95 type 2 diabetic and overweight or obese patients with hemoglobinA1c (HbA1c) ≤ 8% on metformin monotherapy [151]. Individuals were randomized into two groups to receive additional liraglutide or to remain on metformin. EAT thickness significantly decreased only in the liraglutide group, in association with BMI and HbA1c, suggesting that the favorable cardiometabolic effects of liraglutide may be mediated also by modulatory effects on EAT. Morano et al. demonstrated that both liraglutide and exenatide, another GLP-1RA, induce, even after a short treatment, a redistribution of adipose tissue deposits, possibly contributing to a better cardiovascular risk profile in T2DM patients [152].

Recently, the effects on EAT of semaglutide and dulaglutide, two newer long-acting GLP-1RAs, were investigated in a 12 week, controlled, parallel study performed in 80 subjects with T2DM and obesity. Specifically, patients received either semaglutide, up to 1 mg subcutaneous weekly, or dulaglutide, up to 1.5 mg subcutaneous weekly, in addition to their usual medication regimens. The Control group was represented by 20 subjects with T2DM and obesity treated with diet and metformin. EAT thickness significantly decreased in both the semaglutide and dulaglutide groups after 12 weeks, in a dose-dependent manner, while there was no EAT reduction in the metformin group [153]. 

GLP-1R/GIPR dual agonists, such as tirzepatide, are a new class of drugs of great interest, because they have been shown to obtain greatly significant reductions in body weight in both T2DM and obese patients [154]. It will be very important to evaluate the effects of these drugs on distinct adipose tissue depots in obesity.

Sodium-glucose co-transporter 2 inhibitors (SGLT2i) are oral anti-diabetic agents able to reduce hyperglycemia in T2DM patients by increasing urinary glucose excretion. 

As evidenced by multiple cardiovascular outcome trials, SGLT2i reduced the risk of cardiovascular death or hospitalization due to HF in patients with T2DM and an elevated risk of CVD [155]; these effects were observed also in patients with HF regardless of the presence or absence of diabetes [156].

In 13 T2DM patients with T2DM, canagliflozin treatment for 6 months was associated with a significant decrease in HbA1c (from 7.1 ± 0.5% to 6.7 ± 0.6%) and EAT thickness (from 9.3 ± 2.5 to 7.3 ± 2.0 mm), along with a trend of decreasing VAT and SAT area. No association was found between any of these changes [157]. 

In 40 T2DM patients with coronary artery disease, the effect of a 6 month treatment with dapagliflozin on EAT volume and metabolic parameters (including HbA1c, tumor necrotic factor-α (TNF-α), and plasminogen activator inhibitor-1 (PAI-1) levels) was investigated. Treatment with dapagliflozin was able to improve systemic metabolic parameters and to decrease both body weight and EAT volume, thus contributing to CV risk reduction. In addition, changes in EAT volume and body weight, but also in TNF-α levels, showed a significantly positive correlation [158].

These data were confirmed in a 24 week, randomized, double-blind, placebo-controlled clinical trial in T2DM and obese patients. In the dapagliflozin group, EAT decreased by 20% from baseline to 24 weeks, by 15% after 12 weeks, and by 7% between 12 and 24 weeks, respectively, whereas in the metformin group, there was a significant but smaller EAT reduction. There was no statistically significant correlation between EAT and body weight changes, thus suggesting that dapagliflozin causes a rapid and significant EAT reduction that could be independent of weight loss [159].

Recently, Masson et al. performed a very interesting systematic review and meta-analysis of SGLT2i therapy on T2D patients, confirming all of these data on changes in EAT [160]. Therefore, EAT represents a measurable cardiovascular risk factor and a potential modifiable therapeutic target for SGLT2i.

### 3.3. Bariatric Surgery

Bariatric surgery, including Roux-en-Y gastric bypass (RYGB) and sleeve gastrectomy, is indicated in the treatment of severe morbid obesity and obesity-related metabolic complications, since it is shown to be superior to intensive medical therapy, with long-lasting benefits for weight loss, prevention of metabolic diseases, and improvement of quality of life, attributed to weight reduction, reduced inflammatory cell infiltration into the adipose tissue, increased adiponectin levels, and decreased lipolysis and adipocyte cell size [9,13].

Multiple studies have been carried out to evaluate changes in body tissue composition with obesity surgery, with a specific focus on different fatty acid depots. A prospective, non-randomized, single-center cohort study analyzed SAT, VAT, and skeletal muscle with whole-body magnetic resonance imaging in 31 patients with laparoscopic sleeve gastrectomy or RYGB preoperatively, at 3 and 12 month follow-up visits [161]. Patients after bariatric surgery showed a significant reduction in both subcutaneous adipose tissue and VAT. Skeletal muscle was lost only during the first 3 months, and at 12 months, there was no difference between laparoscopic sleeve gastrectomy and RYGB for relative changes in BMI or body tissue composition.

A recent study aimed to determine whether parameters associated with adipose tissue biology, including adipocyte density and the circulating concentrations of markers of adipose tissue dysfunction, might predict cardiovascular risk modification after metabolic surgery in 40 patients with morbid obesity, hypertension, and diabetes mellitus [162]. The extent of cardiovascular damage was estimated using flow-mediated dilation and carotid intima media thickness, which were measured during the 9 months following metabolic surgery. A significant reduction in cardiovascular risk was associated with lower vascular endothelial growth factor-A concentration, low adipocyte density in VAT, low infiltration with CD68+ cells, and higher concentrations of lipid peroxidation markers and malondialdehyde. All of these characteristics might represent useful predictors of the reduction in cardiovascular risk following metabolic surgery.

Willens et al. retrospectively reviewed clinical data and echocardiograms of 23 patients with severe obesity who had echocardiograms recorded before and after bariatric surgery. At baseline, patients had increased epicardial fat compared with normal-weight controls matched for age, gender, and ethnicity. In addition, epicardial fat thickness was associated with the patient’s initial weight in severely obese patients. Patients lost an average of 40 +/− 14 kg after surgery, and epicardial fat thickness significantly decreased from 5.3 +/− 2.4 to 4.0 +/− 1.6 mm (*p* = 0.001), thus suggesting that measuring epicardial fat thickness using echocardiography may be useful to monitor visceral fat loss with weight reduction therapies [163].

The influence of significant weight loss following bariatric surgery was evaluated not only on epicardial fat thickness, but also on carotid intima media thickness, since they represent independent predictors of subclinical atherosclerosis [164]. Both epicardial fat thickness and carotid intima media thickness significantly decreased in 6 months after laparoscopic sleeve gastrectomy surgery. In addition, delta carotid intima media thickness was found to be significantly correlated with delta epicardial fat thickness, delta BMI and delta systolic blood pressure, thus suggesting that early atherosclerotic structural changes may be reversed or improved by sustained weight loss after bariatric surgery in asymptomatic obese patients. Specifically, delta values were obtained by subtracting sixth month values from the baseline values.

A recent comparative study was carried out to evaluate the changes over time of the visceral adipose index, a novel sex-specific index based not only on anthropometric variables, but also on lipid parameters and linked to 10 year cardiovascular risk, in patients who underwent laparoscopic sleeve gastrectomy or RYGB [165]. This study showed that bariatric surgery, independently of the type of surgical procedure, decreased CVD risks due to weight loss and improvement of lipid parameters. Hence, the visceral adipose index could be a useful tool to better identify eligible patients for bariatric surgery and to determine the success of surgery.

## 4. Conclusions

Obesity represents a multifactorial disease that has reached epidemic proportions. Distinct adipose tissue anatomical locations, including both remote adipose tissue depots (intra-abdominal, hepatic, skeletal, pancreatic, renal and mesenteric fat) and cardiac adipose tissue, particularly epicardial adipose tissue, become dysfunctional in the context of obesity, and are held responsible for comorbidities of obesity, including CVD, through multiple molecular mechanisms, mainly the release of proinflammatory cytokines and ROS. Therefore, it is critical to identify strategies capable of reducing cardiovascular risk by modulating adipose tissue mass, distribution, and function. Lifestyle modifications through restriction of food intake and increased energy expenditure, administration of GLP-1 receptor agonists or SGLT2 inhibitors, and bariatric surgery are among the most effective strategies to date to reduce cardiovascular risk by impacting adipose tissues. However, a deeper understanding of the molecular mechanisms responsible for the increase in cardiovascular risk in obese patients remains of primary importance to design novel pharmacological and non-pharmacological strategies to effectively address the CVD burden of obesity.

## Data Availability

Not applicable.
